# Atypical hemolytic uremic syndrome precipitated by thyrotoxicosis: a case report

**DOI:** 10.1186/s12887-020-02082-0

**Published:** 2020-04-17

**Authors:** Ling Hou, Yue Du

**Affiliations:** Pediatric Nephrology Department, Shengjing Hospital of China Medical University, No.36 Sanhao Street Heping District, Shenyang City, 110004 Liaoning Province China

**Keywords:** Atypical hemolytic uremic syndrome, Autoimmune thyroid disease, Therapeutic plasma exchange, Thyrotoxicosis, Thrombotic microangiopathy, Case report

## Abstract

**Background:**

Autoimmune thyroid disease (AITD) has a complex pathogenesis and is associated with the development of autoimmunity against the thyroid. Graves’ disease and Hashimoto’s thyroiditis are the two main types of AITD, and they are characterized by thyrotoxicosis and hypothyroidism, respectively. Atypical hemolytic uremic syndrome (aHUS) is a rare disease, presenting with microangiopathic hemolytic anemia, thrombocytopenia, and acute kidney injury. aHUS is caused by dysregulation of the alternative complement pathway, and its co-existence with AITD is rare.

**Case presentation:**

We report the case of a 12-year-old girl with recent onset thyrotoxicosis. She was first treated with propylthiouracil for 2 months and then developed AITD presenting as abrupt-onset thrombocytopenia, acute kidney injury, and microangiopathic hemolytic anemia. Thyroid function tests favored hyperthyroidism, with increased free T4 and free T3 levels and a very low level of thyroid-stimulating hormone (TSH). We suspected aHUS, and the patient’s condition responded dramatically to therapeutic plasma exchange (TPE) with disease remission. She experienced recurrent aHUS after subsequently receiving methimazole for 1 month, and in the recurrent episode, her condition responded again to TPE and concomitant glucocorticoids. She achieved euthyroidism with thiamazole ointment treatment, without aHUS recurrence during the 6-month follow-up. Mycophenolate mofetil was administered to manage proteinuria after 3 months of treatment with the steroid and angiotensin-converting enzyme inhibitor.

**Conclusions:**

The coexistence of aHUS and AITD in this case is likely more than coincidence, because both are autoimmune in origin. aHUS is associated with a high mortality without appropriate therapy, and treatment with TPE and adjunct immunosuppressants can be helpful.

## Background

Autoimmune thyroid disease (AITD) represents a group of diseases caused by the production of autoantibodies against the thyroid. Examples include Graves’ disease and Hashimoto’s thyroiditis, and the presentations range from hyperthyroidism to hypothyroidism. Atypical hemolytic uremic syndrome (aHUS) is a rare form of thrombotic microangiopathy, characterized by microangiopathic hemolytic anemia, thrombocytopenia, and acute kidney injury. The pathogenesis of aHUS includes congenital mutations involving complement regulatory factors or complement per se, or the development of autoantibodies against factor H, which is pathogenic due to dysregulation of the alternative complement pathway. However, the coexistence of aHUS and AITD has been rarely reported in the literature. We herein report a case of aHUS in a child with AITD.

## Case presentation

A 12-year-old Chinese Han girl complained of nose bleeds, vomiting, fatigue, and passing reddish urine for 3 days. She did not have fever, diarrhea, or any neurological symptom, and her blood pressure was normal. She did not have a history of organ transplantation or hematopoietic stem cell transplantation. She had recent onset hyperthyroidism due to AITD, with initial presentation of marasmus Cachexia and exophthalmos. Her blood tests results were as follows: increased free T3 and T4 levels (T3 7.65 pmol/L, [reference 2.63–5.71 pmol/L] and T4 22.17 pmol/L [reference 9.01–19.05 pmol/L]), a decreased thyroid-stimulating hormone (TSH) level (0.0006 μIU/mL [reference 0.30–4.8 μIU/mL]), an elevated anti-Tg antibody at 59.55 IU/mL (reference 0–5.61 IU/mL), an increased anti-TPO antibody at 51.90 IU/mL (reference 0–4.11 IU/mL), and an increased TSH receptor antibody at 24.760 IU/L (reference 0–1.75 IU/L). Thyroid ultrasound in a local hospital showed diffuse thyroid lesions consistent with hyperthyroidism. She was treated with propylthiouracil (0.1 g thrice daily for 1 month, 0.1 g twice daily for 1 month) since her previous hospitalization for AITD.

Post treatment laboratory tests showed severe thrombocytopenia (10–13 × 10^9^/L), anemia (48–80 g/L hemoglobin), and microangiopathic hemolytic anemia based on the combination of a negative direct antiglobulin test, a reticulocyte percentage of 6.6%, a lactate dehydrogenase (LDH) concentration of 688 U/L, a total bilirubin concentration of 113 μmol/L, and an unconjugated bilirubin concentration of 100.5 μmol/L. There were schistocytes and thrombocytopenia on peripheral blood smear. Tests showed that ‘A disintegrin and metalloproteinase with a thrombospondin type 1 motif member 13’ (ADAMTS13) activity was 100% (normal range 40–130%), anti-ADAMTS13 antibody was negative, and anti-CFH antibody was positive. A thyroid function test showed hyperthyroidism, with increased free T3 and T4 levels (T3 7.92 pmol/L [reference 2.63–5.71 pmol/L] and T4 21.05 pmol/L [reference 9.01–19.05 pmol/L]) and a decreased thyroid-stimulating hormone (TSH) level (0.0028 μIU/mL [reference 0.30–4.8 μIU/mL]). In addition, blood tests showed normal levels of amylase (58 U/L [reference 40–129 U/L]), lipase (23.5 U/L [reference 0–60 U/L]) and blood homocysteine (30.41 μmol/L [reference 0–15 μmol/L]), while renal function tests showed that her blood urea nitrogen (BUN) and serum creatinine concentrations were 29.57 mmol/L (reference 2.5–7.2 mmol/L) and 153.3 μmol/L (reference 45–84 μmol/L), respectively, indicating acute kidney injury. Tests for anti-nuclear antibody (ANA), anti-neutrophil cytoplasmic antibody (ANCA), and anti-cardiolipin antibody (ACA) were all negative, but the patient’s C3 and C4 levels were slightly decreased(C3 0.634 g/L [reference 0.74–1.4 g/L] and C4 0.0825 g/L [reference 0.12–0.36 g/L]). Stool culture was negative.

Chest and abdominal computed tomography (CT) scanning did not reveal any abnormalities, and the results of bone marrow aspiration were also normal. We suspected that this patient had aHUS. Daily therapeutic plasma exchange (TPE) for 2 days resulted in an excellent clinical and laboratory response, with normalization of the platelet count (182 × 10^9^/L), renal function, and LDH concentration. She did not have proteinuria (urinalysis showed no proteinuria). Because propylthiouracil may trigger aHUS, we changed the propylthiouracil to methimazole (morning 5 mg and evening 2.5 mg daily).

**Fig. 1:**
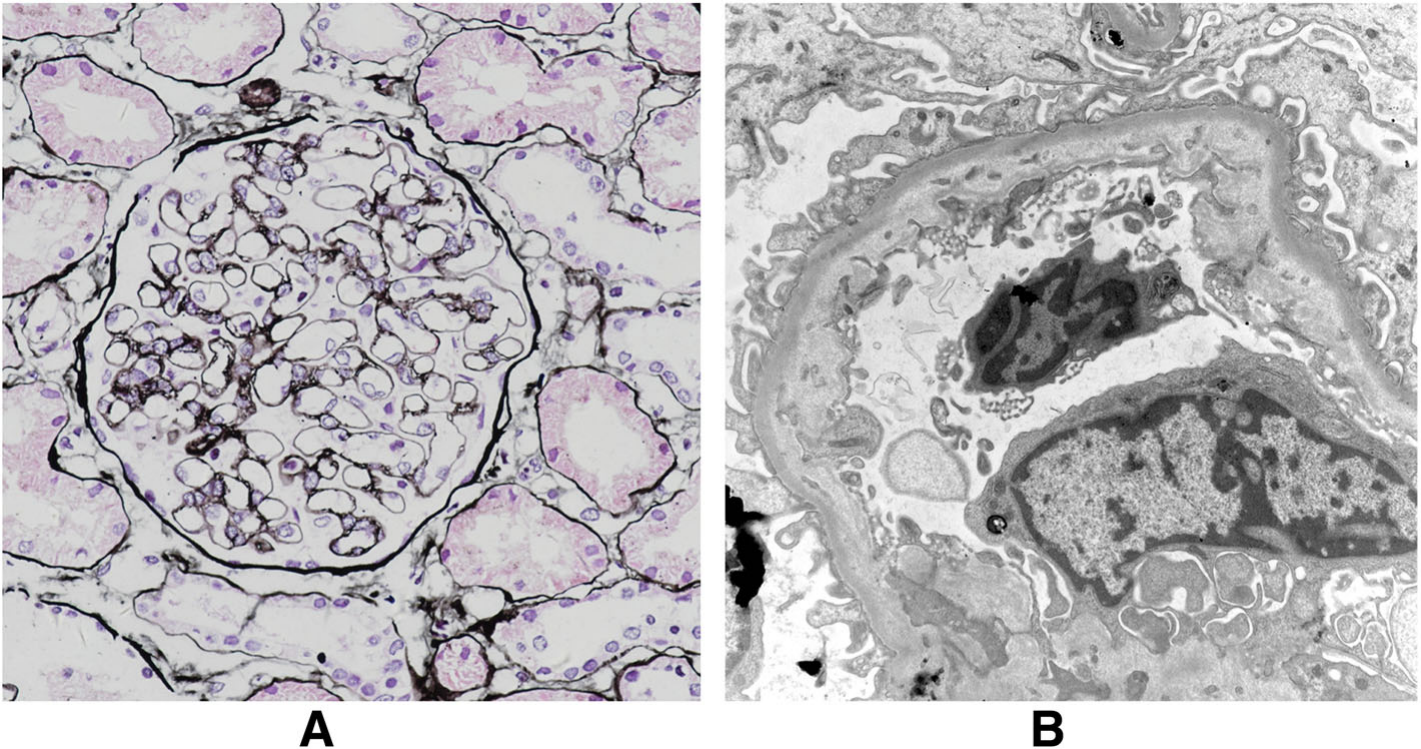
**a** Light microscopy showed mild segmental mesangial proliferation, while PASM staining showed focal segmental chain-ring change over glomerular basement membrane and tubular epithelial swelling with 5% interstitial edema. Immunofluorescent staining (× 400) showed that the tissue was IgG (−), IgA (−), IgM (+/−), C3 (−), C1q (−), and fibrinogen (−). **b** Electron microscopy (× 10,000) showed inner thin layer of glomerular capillary basement membrane thickening and endothelial swelling, without any electron dense deposits

One month later, the patient was re-admitted with bleeding spots over her upper extremities for 1 day, without fever, diarrhea, or nervous system symptoms. Laboratory tests showed that thrombocytopenia (47 × 10^9^/L) and anemia (101 g/L) recurred (hemoglobin, 136 g/L 10 days ago), and her LDH (1002 U/L), total bilirubin (95.8 μmol/L), and indirect bilirubin (85.0 μmol/L) levels had all increased, accompanied by acute kidney injury (BUN 14.21 mmol/L and creatinine 106.2 μmol/L). Thyroid function results showed subclinical hyperthyroidism (normal free T3 [3.63 pmol/L] and T4 [13.71 pmol/L] but low TSH [0.0133 μIU/mL]). Repeat examination for ANA, ANCA, and ACA expression again returned normal results. The above results supported the diagnosis of recurrent aHUS. Therefore, we re-administered TPE once every other day for two sessions, and the response was good. Genetic tests based on whole exome sequencing identified a *CFHR1*c.641 T > A (p.I214N) missense mutation, which was also found in the test of her asymptomatic father, although this mutation was thought to be harmless. Renal biopsy findings were compatible with HUS (Fig. 1a and b). The patient achieved euthyroidism with thiamazole ointment treatment, and aHUS did not recur during the 6 months of follow-up. Proteinuria was 2.77 g/d after the second episode of HUS, and we started steroid treatment (prednisone 60 mg/day with tapering by 5 mg every 2 weeks). Because proteinuria recurred (1.58 g/d) after 3 months of steroid and angiotensin-converting enzyme inhibitor treatment, we administered mycophenolate mofetil (0.75 g and 0.5 g daily in the morning and in the evening, respectively). Her daily proteinuria had decreased to 0.5 g after 6 months of follow-up.

## Discussion and conclusion

The diagnosis of aHUS in this case was made based on clinical features and the response to treatments. ADAMTS13 activity and anti-ADAMTS13 antibody were both normal, with positive anti-CFH antibody. aHUS is a disorder involving dysregulation of the complement system, leading to capillary endothelial damage and microangiopathic hemolytic anemia. aHUS can be secondary to rheumatologic disorders, including systemic lupus erythematosus, renal crisis of scleroderma, antiphospholipid syndrome, vasculitis, etc. [1]. We did not find evidence supporting the above diagnoses, since our patient didn’t have compatible symptoms and the test results of ANA, ANCA and ACA were all negative. About 5–10% of aHUS cases are caused by autoantibodies against complement H factor [2], and aHUS may also be an autoimmune disorder. Based on this patient’s disease course, excellent treatment response to TPE, and the absence of a pathogenic mutation on genetic analysis, we favored that she might have aHUS caused by autoantibodies against the complement system, leading to excessive activation of the complement pathway and microangiopathic hemolytic anemia. However, more evidence is needed to exclude the possibility that she had other genetic causes of HUS or HUS caused by activated complements.

AITD is an organ-limited autoimmune disorder, with the thyroid as the target organ. It is caused by the destruction of thyroid tissue with anti-thyroglobulin antibody, anti-thyroid peroxidase antibody, or anti-thyrotropin receptor antibody [3]. AITD is frequently accompanied by systemic lupus erythematosus (SLE), but reports of AITD occurring with TTP are rare [4–9]. TTP and aHUS both belong to the spectrum of thrombotic microangiopathy (TMA), sharing an autoantibody-related mechanism. Consequently, we believe that in our case, aHUS might have been pathogenetically associated with the patient’s background AITD.

In addition, anti-thyroid medications, including propylthiouracil and methimazole, have hematologic side effects such as agranulocytosis, anemia, and thrombocytopenia. Propylthiouracil has been reported to cause ANCA-associated vasculitis, and we presume that the drug may be another potential precipitator of aHUS. Several reasons support this presumption: first, our patient discontinued propylthiouracil and methimazole after TPE and received thiamazole ointment and steroid for AITD, and aHUS did not recur thereafter. Second, the propensity of propylthiouracil to induce autoantibody production may extend beyond ANCA to include those against the complement system, although this theory still needs clarification.

The patient in the present case presented with hyperthyroidism when her first episode of aHUS occurred, and she had subclinical hyperthyroidism during her second episode of aHUS, followed by an aHUS-free and euthyroid status thereafter. It is known that hyperthyroidism can cause endothelial dysfunction and a prothrombotic tendency, and hyperthyroidism may have been an important predisposing factor for aHUS in this patient. Based on the above reasons, we believe that in this patient, the development of aHUS was pathogenetically linked to her AITD due to the shared autoimmunity pathogenesis, hyperthyroidism-related vascular injury, and use of anti-thyroid medications. The renal survival and overall survival rates among patients with aHUS are poor if appropriate treatments are not given early [10]. Therefore, earlier diagnosis and timely TPE administration are important critical to improving patients’ prognosis, as was shown in our case.

Eculizumab is a monoclonal antibody against C5, which acts on the terminal of complement activation and stops the cleavage of C5, thereby blocking the formation of membrane attack complex and effectively quenching complement actions. It is effective for treating both hereditary and acquired aHUS in children, especially for those with a poor prognosis due to ineffective PE or the dependence on PE [11]. Eculizumab was first administered to patients with aHUS in 2009, and has been approved later for the treatment of aHUS in United States and European Union but yet to be approved for use in mainland China.

## Data Availability

The datasets used during the current study available from the corresponding author on reasonable request.

## Abbreviations

AITD: Autoimmune thyroid disease
aHUS: Atypical hemolytic uremic syndrome
TSH: Thyroid-stimulating hormone
TPE: Therapeutic plasma exchange
ADAMTS13: A disintegrin and metalloproteinase with a thrombospondin type 1 motif member 13′
BUN: Blood urea nitrogen
ANA: Anti-nuclear antibody
ACA: Anti-cardiolipin antibody
ANCA: Anti-neutrophil cytoplasmic antibody
CT: Computed tomography
SLE: Systemic lupus erythematosus
TTP: Thrombotic thrombocytopenic purpura
TMA: Thrombotic microangiopathy
